# Copy Number Variants in the 11p15.5 Associated Imprinting Disorders: An Attempt to Establish a Genotype–Phenotype Correlation

**DOI:** 10.1111/cge.70139

**Published:** 2026-01-13

**Authors:** Anastasia Maria Licata, Elke Botzenhart, Katja Kloth‐Stachnau, Thomas Eggermann

**Affiliations:** ^1^ Center for Human Genetics and Genome Medicine, Medical Faculty RWTH University Aachen Aachen Germany; ^2^ Institute for Clinical Genetics, Klinikum Stuttgart Stuttgart Germany; ^3^ amedes MVZ für Humangenetik Hamburg GmbH Hamburg Germany

**Keywords:** 11p15.5 copy number variants, Beckwith–Wiedemann syndrome, genotype–phenotype correlation, Silver–Russell syndrome

## Abstract

Copy number variations (CNVs) affecting the imprinted regions in 11p15.5 (imprinting centre 1 and 2/IC1, IC2) account for more than 2% of the molecular disturbances in Beckwith‐Wiedemann and Silver–Russell syndrome (BWS, SRS) and are associated with a recurrence probability of up to 50%. However, their clinical impact can be challenging to estimate, as it depends on the type of imbalance, the parental origin of the affected allele, its size and genomic content. As a result, a genotype–phenotype correlation of 11p15.5 alterations is still missing, at least for CNVs affecting only parts of the IC1 or IC2. By comprehensively summarising all published CNVs within 11p15.5 and the available clinical data of their carriers, we aim to further delineate a correlation of these disturbances with BWS and SRS features. In fact, consistent correlations could be delineated only for duplications including either both the telomeric and centromeric regions or complete gains of one of them. In contrast, CNVs encompassing only parts of these regions lead to heterogeneous phenotypes. In summary, our literature review provides support for pathogenicity assessment of CNVs in 11p15.5 as basis for genetic counselling. However, this dataset underlines the need for further research to enlighten the molecular complexity of this region and to better understand the regulation of genomic imprinting mechanisms in 11p15.5.

## Graphical abstract

Copy number variations (CNVs) in 11p15.5 account for more than 2% of the molecular disturbances in the imprinting disorders Beckwith–Wiedemann and Silver–Russell syndrome. Their size and gene content vary, and therefore the impact on the phenotype is variable. Based on published data from > 220 carriers, an overview of the pathogenicity of 11p15.5 CNVs is provided as a basis for genetic counselling.

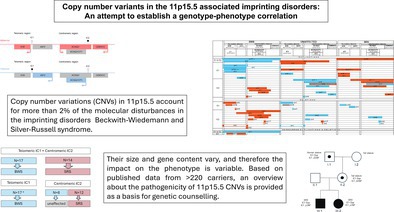

## 1 Introduction

Molecular alterations disrupting one or both imprinting centres (IC1, IC2) on chromosome 11p15.5 are associated with two clinically opposite growth disorders, Beckwith–Wiedemann syndrome (BWS) and Silver–Russel syndrome (SRS). These disturbances comprise imprinting defects (gain or loss of methylation; GOM/LOM), uniparental disomy (UPD), single nucleotide variants (SNVs), and copy number variants (CNVs).

The most frequently observed pathogenic changes in both BWS and SRS are imprinting defects (> 60%), followed by UPD (upd(11)pat in BWS, 16% upd(7)mat in SRS [1]). CNVs contribute to up to 2.5% of molecularly diagnosed BWS and SRS patients [1] and their identification is relevant for genetic counselling as the recurrence risk is increased in these families and the clinical outcome depends on the parental origin and genomic content of the affected allele. In contrast, recurrence risk is low for imprinting defects and UPDs.

**FIGURE 1:**
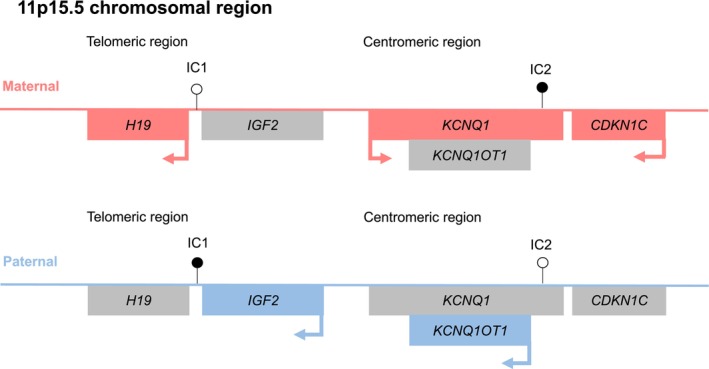
The imprinted regions in 11p15.5. (filled/empty lolly pops, methylated/unmethylated DMR; blue, paternal allele; red, maternal allele; sizes and positions are not drawn to scale).

The telomeric IC1 and the centromeric IC2 in 11p15.5 regulate the monoallelic expression of imprinted genes (Figure 1), e.g., *IGF2* and *H19* (IC1) and *KCNQ1*, *KCNQ1OT1* and *CDKN1C* (IC2). In fact, pathogenic SNVs of *CDKN1C* on the maternal allele are diagnosed in up to 5% of sporadic BWS cases [2]. In a small subset of SRS patients, pathogenic SNVs in *CDKN1C* [3] on the maternal and *IGF2* variants on the paternal allele can be identified [4, 5].

Pathogenicity classification, genetic counselling, and phenotype prediction of CNVs and other structural variants (SVs) affecting the imprinted 11p15.5 regions are challenging as SVs affecting nearly the same genomic region but of slightly different size can lead to different clinical outcomes. Accordingly, their classification requires consideration of the type of imbalance (e.g., gain or loss of genomic material), parental origin of the affected allele, size, functional content and methylation status of the affected differentially methylated region (DMR).

Since the first report on a de novo duplication in a BWS patient in 1983 [6], more than 200 carriers of SVs within the 11p15.5 region have been published (Table S1). By compiling all SVs within 11p15.5 reported until August 2025 and by including four new cases from our cohort, this study aims to delineate genotype–phenotype correlations for 11p15.5 SVs with a focus on CNVs as the basis for directed genetic counselling.

## 2 Methods

Our study includes all structural variants (SVs) affecting the imprinted regions in 11p15.5 and associated with clinical features of BWS or SRS published from 1983 to August 2025. The publications were identified by a systematic search through the PubMed database (https://www.ncbi.nlm.nih.gov/pubmed/) using the following MeSH terms: (‘deletion’ OR ‘duplication’ OR ‘translocation’ OR ‘structural variants’ OR ‘copy number variations’) AND (‘11p15.5’) AND (‘Beckwith–Wiedemann‐syndrome’ OR ‘Silver–Russell‐syndrome’). The final PubMed search was conducted on 31.08.2025. In addition, the DECIPHER database was checked for cases with gains or losses in the region (https://www.deciphergenomics.org/).

For breakpoint delineation of the SVs the detection methods used in the original study were considered. Whereas array‐based analyses, (methylation‐specific) multiplex ligation probe‐dependent analysis ((MS‐)MLPA) and short tandem repeats (STR) did not allow a precise breakpoint mapping, this was possible for cases characterised by sequencing (Sanger‐Sequencing, short read sequencing, long read sequencing). Publications with insufficient or inconsistent information about the breakpoint positions were excluded from the study (Table S1, Figure S1).

All genomic coordinates were converted into GRCh38/hg38. Deletions and duplications smaller than 50 bp were not considered according to the standard definition of CNVs [7]. The physical positions of the telomeric IC1 and the centromeric IC2 genomic regions were based on Monk et al. [8].

Due to the heterogeneity of the available clinical data in the patient cohort, the suggested clinical scoring systems for BWS or SRS [9, 10] could not be applied. Patients were therefore classified as patients with ‘BWS or SRS features’ by growth‐related features, i.e., prenatal and/or postnatal overgrowth in BWS and growth restriction in SRS.

For new duplication cases from our own cohort, MS‐MLPA was performed according to the manufacturer's instructions, using the ME030 probe mix (MRC‐Holland, Amsterdam/Netherlands). Results were analysed using Coffalyser.Net. These patients gave informed consent for participation, and the study was approved by the ethical committee of the Medical Faculty of the RWTH Aachen (EK303‐18).

## 3 Results

**FIGURE 2:**
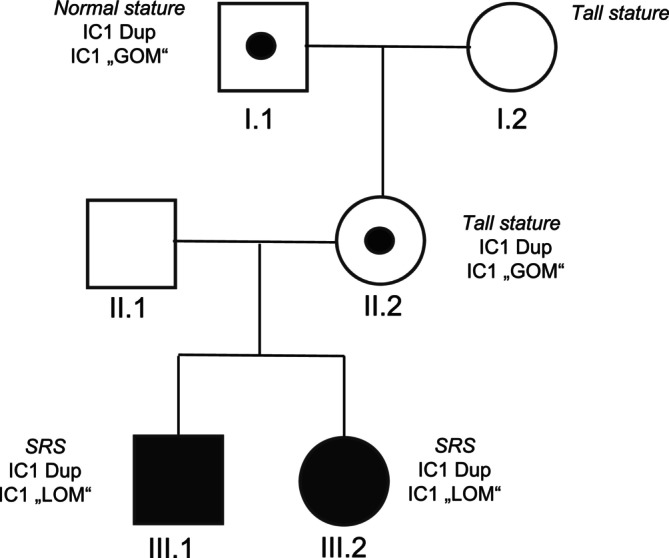
Pedigree of a family with an IC1 duplication, originally published in [11](Figure 3: line 8; Table S1: family of patient 97). Whereas the children (III.1/0.2) carrying the maternally inherited duplication of the IC1 exhibited SRS, the mother (II.2) showed gain of the paternal allele and was reported as tall. However, by segregation analysis it could be shown now that duplication of the paternal allele is not associated with clinical features but the tall stature of the mother occurred independently.

**FIGURE 3:**
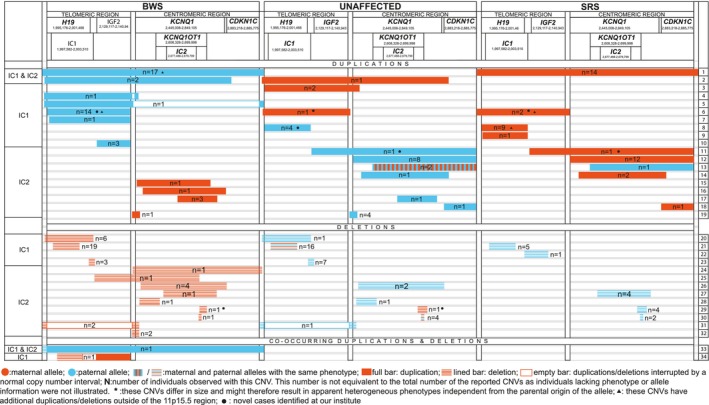
Overview of the published CNVs within the telomeric and centromeric imprinted regions in 11p15.5. The sizes of the CNVs and regions are not drawn to scale. The CNVs are grouped according to the associated phenotypes: BWS (Beckwith–Wiedemann Syndrome associated features), SRS (Silver–Russel Syndrome associated features) or unaffected. Furthermore, the cases have been discriminated into deletions, duplications and co‐occurring duplications and deletions. The numbers refer to the number of individuals reported to carry the respective CNVs. Translocations are not included in the figure. Information about all cases is provided in Table S1. Transcripts of the coding genes *IGF2*, *CDKN1C*, *KCNQ1* and the non‐coding genes *H19* and *KCNQ1OT1* were based on the MANE transcripts. Genomic breakpoints of the CTCF binding sites were based on AF125183 (https://www.ncbi.nlm.nih.gov/nuccore/AF125183). All genomic coordinates refer to GRCh38/hg38. Each molecular subgroup is provided with a line number on the right side of the illustration. The same line number is also provided in Table S1, allowing the reader to cross‐reference the cases between Figure 3 and Table S1, where precise breakpoints for each case are shown. It has to be noted that not all cases listed in Table S1 could be included in Figure 3. CNVs with unknown parental allele, missing clinical data or phenotypes other than BWS/SRS were not shown in Figure 3.

We report on four new cases carrying CNVs within 11p15.5, one of which involves the maternal grandfather of a patient recently published by our group (patient 3 from [11]; Figure 2: I.1; Figure 3: line 8). The new cases consist of four duplications encompassing the telomeric region IC1 (*n* = 2) or the entire centromeric region IC2 (*n* = 2).

The two telomeric duplications affected the paternal allele. One encompassed the entire IC1 region and resulted in BWS features (Figure 3: line 6). The second duplication (maternal grandfather of Pat. 3 from [11]; Figure 3: line 8) affected IC1 and *H19*, excluding *IGF2*, thereby leading to no clinical phenotype.

The two centromeric duplications additionally included the *IGF2* gene (Figure 3: line 11). These led either to SRS in case of maternal inheritance or an unaffected phenotype in case of paternal transmission.

Out of 78 selected publications, 58 provided sufficient information to consider the reported cases. In these papers, we identified 224 cases, out of which 118 were index patients (Figure S1). Among the index cases, 56 were identified as ‘familial cases’ and 62 occurred sporadically, described as ‘single cases’ as follows:

In total, considering both the 224 cases reported in the literature and the 4 novel cases identified at our institute, we documented 228 structural variants (SVs) within 11p15.5. These comprised single duplications (*n* = 118), single deletions (*n* = 99), interrupted duplications (*n* = 2), interrupted deletions (*n* = 3), co‐occurring duplications and deletions (*n* = 2), and balanced chromosomal translocations (*n* = 4) (Table S1).

Among the 228 cases documented in this study, cases with additional genomic imbalances affecting other chromosomes (*n* = 12) or regions outside the imprinting cluster on chromosome 11 (*n* = 2) were present. In these cases the contribution of the additionally affected chromosomal regions to the clinical phenotype has to be considered.

The most frequent duplications included large gains of more than 1 Mb encompassing both the entire telomeric and centromeric regions as well as isolated duplications of the complete telomeric region or the complete centromeric region.

As the clinical association between duplications including both centromeric and telomeric imprinted regions and BWS/SRS features is well established, these cases are often not published. Therefore, the number of patients with these large duplications ascertained in our study does not reflect the actual number of patients with this genetic constitution.

**FIGURE 4:**
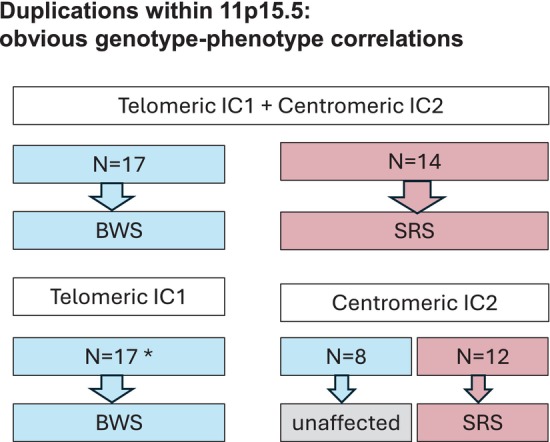
Overview on the 11p15.5 duplications with obvious genotype–phenotype correlations. (For further details: See Figure 3; blue, paternal inheritance; red, maternal inheritance; BWS, Beckwith–Wiedemann syndrome; SRS, Silver–Russell syndrome; *Two interrupted IC1 duplications were included (See Figure 3: lines 4, 5)).

For duplications comprising both imprinting domains, a consistent genotype–phenotype correlation could be delineated (Figure 3: line 1; Figure 4). The same was observed for smaller duplications affecting the *H19* and IC1 subregions (Figure 3: lines 8, 9), partial segments of *KCNQ1* (Figure 3: line 19), or the entire *IGF2* locus (Figure 3: line 10; Figure 4).

The most frequently observed deletion affected the telomeric region spanning parts of both the IC1 and *H19* loci (Figure 3: line 21) followed by deletions affecting only the IC1 locus (Figure 3: line 23) and the entire IC1 and *H19* loci (Figure 3: line 20). However, the numbers were too small to delineate genotype–phenotype correlations.

Deletions encompassing the IC2 region were very heterogeneous in size (varying from 132 bp to 600 kb) and breakpoint positions. Therefore, genotype–phenotype correlation could not be delineated for this region.

Balanced translocations were reported in four cases [12, 13] (Table S1). These resulted in the disruption of the *IGF2* gene or of the *KCNQ1* gene.

## 4 Discussion

CNVs affecting the imprinted regions in 11p15.5 significantly contribute to the molecular spectrum of BWS and SRS (up to 2.5% of patients [1]), with a preponderance of duplications. Previous studies and case reports illustrate the molecular and clinical heterogeneity of 11p15.5 disturbances (Figure 3, Table S1) [14, 15]. Accordingly, evaluating the pathogenicity of CNVs and other SVs in 11p15.5 is often challenging in respect to classify the variant as disease causing as well as to correlate or even predict the clinical outcome (i.e., in prenatal diagnosis).

To facilitate the evaluation of the pathogenicity of 11p15.5 SVs, we conducted a literature search for these cases. After reevaluating the available molecular and clinical data of 228 SV carriers, we could identify variants with a consistent genotype–phenotype correlation (Figure 4), as well as variants associated with heterogeneous clinical phenotypes. In addition, our characterisation of 11p15.5 SVs provides new insights in the functional role of 11p15.5 regulatory elements and their interaction.

As the number of other SVs than CNVs (e.g., balanced translocations) is too small to delineate consistent genotype–phenotype correlations, in the following only CNVs are discussed. Additionally, the carriers of 132 bp deletions spanning *KCNQ1* and *KCNQ1OT1* (*n* = 7; Figure 3: line 30) have already been reviewed by [16] and are not further discussed in this paper.

### 4.1 CNVs of Both Imprinted Regions in 11p15.5

Large duplications comprising the complete telomeric (IC1) and centromeric (IC2) imprinted regions in 11p15.5 are consistently associated with BWS in case the paternal allele is affected (*n* = 17; Figures 3 and 4: line 1), or with SRS in case of maternal duplications (*n* = 14; Figures 3 and 4: line 1). Though the functional consequence of these duplications has not yet been finally identified, several studies indicate a contribution of both *IGF2* in the telomeric and *CDKN1C* in the centromeric imprinted region of 11p15.5 to the aetiology of BWS and SRS (e.g., BWS: [2], SRS: [3, 5]). In fact, the duplication of the paternal telomeric copy is sufficient to cause a BWS phenotype (see below, Figure 4).

For large duplications of the entire 11p15.15 imprinted regions it has to be considered that additional genes might be affected; therefore, the patients might exhibit features additional to the BWS or SRS phenotypes. This was observed for 10 carriers of paternal duplications spanning the entire IC1 and IC2 regions (Figure 3: line 1; Table S1).

Large CNVs of complete IC1 and IC2 are also listed by DECIPHER (https://www.deciphergenomics.org/) but are not published in context with BWS or SRS and have therefore not been considered.

### 4.2 CNVs Affecting the Telomeric Imprinted Region in 11p15.5—Duplications

A further consistent correlation can be delineated for duplications encompassing the entire telomeric region on the paternal allele (Figure 4; two interrupted IC1 duplications (see Figure 3: lines 4, 5) were included in the total count). These are always associated with BWS (*n* = 15; Figure 3: lines 6, 7).

In contrast, maternal duplications of the entire telomeric region resulted in heterogeneous phenotypes (*n* = 3; Figure 3: line 6). One case was clinically unaffected, whereas two cases were associated with SRS features. These maternal duplications differed significantly in both size and breakpoint localisation (Table S1).

The SRS‐associated duplications [17, 18] were large (600 kb and 1.9 Mb, respectively) and extended both into regions telomeric to *H19* and centromeric to *IGF2*. In contrast, the duplication without phenotypic effect [19] was 300 kb and extended only minimally beyond *H19* and *IGF2* (see Figure 3). By considering the silencing of *IGF2* on the maternal allele, its duplication would not be expected to result in a growth restriction phenotype. However, the observations in these two cases indicate that the breakpoints and sizes of the CNVs play a crucial role for the clinical outcome.

We therefore assume that larger IC1 duplications (> 300 kb) may harbour regulatory elements affecting *IGF2* expression, thereby explaining the SRS features despite the expected maternal silencing. In fact, Jurkiewicz et al. [18] and Demars et al. [20] also suggested a trans‐acting mechanism in which an additional functional copy of *H19* may affect the expression of *IGF2*, thereby resulting in growth restriction.

Two similar maternal duplications additionally including part of *KCNQ1* have been reported without an impact on the phenotype (Figure 3: line 3). Like the IC1 duplication described above with unaffected phenotype, these duplications did not extend significantly into regions telomeric to *H19*.

This may provide further support for our hypothesis that involvement of regions telomeric to *H19* in the context of maternal duplications is necessary for manifestation of the SRS‐related features.

Two cases of interrupted IC1 duplications have been reported (Figure 3: lines 4, 5). These paternal duplications were associated with BWS features and spanned the entire telomeric region. After a segment of normal copy number outside the IC1 region, one duplication continued to the telomeric part of the *KCNQ1* gene [14] (Figure 3; line 4) while the second extended further into a region located centromeric from the *CDKN1C* gene [21] (Figure 3: line 5). These duplicated parts beyond the telomeric region apparently do not affect functionally relevant features as these interrupted duplications clinically correspond to gains of the telomeric region only.

Duplications of the IC1 and *H19* loci, excluding *IGF2*, seem to correlate with SRS features in case the maternal allele is affected (*n* = 10; Figure 3: lines 8, 9), whereas duplications of the paternal allele are not associated with clinical features (*n* = 4; Figure 3: line 8).

Duplications of the paternal *IGF2* gene alone resulted in BWS features in three cases (Figure 3: line 10). This observation is compatible with the function of the gene as a growth promoter. Although maternal *IGF2* duplications have not yet been documented in the literature, current knowledge about the IC1 region suggests that they will not cause an aberrant phenotype as the gene is silenced on the maternal allele.

### 4.3 CNVs Affecting the Telomeric Imprinted Region in 11p15.5—Deletions

The most frequent losses described in 11p15.5 are deletions of the maternal copies of both IC1 and *H19* (*n* = 25; Figure 3: lines 20, 21). These deletions have been associated with BWS features in 25 cases, whereas two cases without clinical signs have been reported [22] (Figure 3: line 21: two of the unaffected carriers; [22]).

Carriers of deletions of the paternal IC1 and *H19* allele showed either SRS features (*n* = 5; Figure 3: line 21) or no clinical signs (*n* = 15; Figure 3: one case of line 20, 14 cases of line 21). The reason for this heterogeneity is unclear as all cases exhibit an IC1 LOM, but it can be postulated that despite involving the same genes, the differences in breakpoint positions might have an impact on regulative elements and chromatin organisation in this region.

We also identified a recurrent small deletion affecting small parts of the IC1 locus (Figure 3: line 23). This CNV appears to be associated with BWS features in case the maternal allele is affected (*n* = 3), whereas the deletion on the paternal allele does not obviously cause clinical features (*n* = 7).

Paternal loss of portions of IC1 and *IGF2* has been reported only once and led to SRS features (Figure 3: line 22).

### 4.4 CNVs Affecting the Centromeric Imprinted Region in 11p15.5—Duplications

Another obvious genotype–phenotype correlation can be delineated for duplications of the entire centromeric 11p15.5 imprinted region (Figure 3: line 12; Figure 4). Maternal duplications (*n* = 12) are associated with SRS features, whereas duplications of the paternal allele (*n* = 8) are observed in healthy individuals.

Three reported duplications encompassing parts of *KCNQ1*, the entire IC2 and *KCNQ1OT1*, and parts of *CDKN1C* [23] (Figure 3: line 14) followed the same phenotype correlation as the entire centromeric duplications: gains of the maternal allele are associated with SRS features (*n* = 2), whereas those of the paternal allele are not correlated with a specific phenotype (*n* = 1).

Two cases with duplications of the entire *CDKN1C* gene [24] (Figure 3: line 18) followed the same pattern as entire centromeric duplications, resulting in SRS features in case the maternal allele is duplicated (*n* = 1) and in an unaffected phenotype when the paternal allele is affected (*n* = 1).

However, two reported cases with duplications of almost the entire centromeric imprinted region (Figure 3: line 13) did not follow the pattern observed for duplications of the entire region (Figure 4). In these cases, the breakpoint positions slightly differed from entire centromeric duplications and included parts of *KCNQ1* and of *KCNQ1OT1*, the complete IC2 locus and the entire *CDKN1C* gene. One paternal duplication of this region led to increased *CDKN1C* expression and therefore to SRS clinical features [25]. This outcome contrasts with the reported unaffected cases carrying duplications of the paternal IC2 allele. Xue et al. [25] postulated that the partial duplication of *KCNQ1OT1* should explain the atypical SRS phenotype as its transcript might be unstable and thereby not inhibit the *CDKN1C* expression, leading to an elevated CDKN1C dosage.

Another case with duplication of the maternal allele of this region did not reveal the expectable SRS features and the authors assumed normal *CDKN1C* expression [24].

We identified five cases with duplications of the maternal centromeric copy excluding *CDKN1C* (Figure 3: lines 15–17). These were associated with BWS features, different from duplications encompassing the maternal *CDKN1C* copy which are mainly associated with SRS phenotypic traits. This clinical outcome cannot be explained by the duplication itself, as the duplicated maternal copy of *KCNQ1OT1* transcript should remain silent and would not inhibit *CDKN1C* expression. All reported BWS cases with this disturbance exhibited LOM of the IC2, suggesting that the duplication interferes with normal imprinting establishment. Demars et al. [20] previously hypothesised (in the context of a partial *KCNQ1* duplication leading to IC2 LOM and BWS) that *cis*‐duplications in *KCNQ1* could alter chromatin architecture, thereby disrupting methylation establishment at IC2. As in all five patients the *KCNQ1* gene was partially duplicated, the BWS phenotype can therefore be explained by the associated LOM at IC2 resulting in silencing of *CDKN1C*.

Five reported duplications of the paternal IC2 copy of similar size and position did not lead to a clinical phenotype (Figure 3: *n* = 1, line 17; *n* = 4, line 19).

### 4.5 CNVs Affecting the Centromeric Imprinted Region in 11p15.5—Deletions

Loss of the entire IC2 region has been reported only once for the maternal allele and resulted in BWS features (Figure 3: line 24).

**FIGURE 5:**
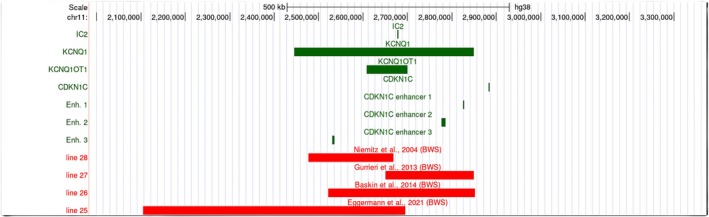
Custom track for the four published deletions within the maternal allele of the IC2 region. All patients showed BWS features. For each deletion, the corresponding line number of Figure 3 is indicated on the left side. The physical position of the *CDKN1C* enhancers were based on [26]. (red, deletions; green, IC2 genes and *CDKN1C* enhancers).

Interestingly, both maternal duplications and deletions of the centromeric region excluding *CDKN1C* led to BWS clinical features (Figure 3: lines 15–17, 25–29, 31–32). The phenotypic outcome of the deletions can be explained either by deleted *CDKN1C* enhancers (*n* = 7 Figure 3: lines 25–28; Figure 5) [21, 27, 28, 29, 30], according to the genomic locations of the *CDKN1C* enhancers [26] or, in case of intact enhancers, by IC2 LOM (*n* = 5; Figure 3: lines 29, 31, 32) [21, 31, 32, 33]. This aberrant IC2 methylation may result from the partial deletion of *KCNQ1* in these cases [34].

Carriers of paternal deletions of the centromeric imprinting region excluding *CDKN1C* (*n* = 11; Figure 3: lines 26–29) showed heterogeneous clinical pictures, with a preponderance of SRS features (*n* = 8 out of 11 cases; Figure 3: lines 27, 29). As all deletions disturbed both *KCNQ1* and *KCNQ1OT1*, the SRS clinical traits might be explained by the lack of paternal *KCNQ1OT1* transcript causing an increased *CDKN1C* expression and resulting in a growth restriction phenotype. However, the lack of SRS features in three carriers (Figure 3: lines 26, 28) might be explained by deletion of *CDKN1C* enhancer elements, which might thereby neutralise the lack of inhibition by KCNQ1OT1.

## 5 Conclusions

The summary of all published cases with gains and losses affecting the two imprinted regions in 11p15.5 illustrates the challenge to delineate a genotype–phenotype correlation. In fact, consistent correlations could be delineated for certain larger duplications (Figure 4), which numerically account for the largest proportion of 11p15.5 CNV carriers, but the majority of the published CNVs show heterogeneous clinical pictures (Figure 3). It can be assumed that the clinical consequence of these CNVs is not only associated with the genes and regulatory regions affected by the disturbance, but presumably also by their impact on spatial chromatin organisation. Our compilation of cases with CNVs in 11p15.5 (Figure 3) should help to classify the pathogenicity of SVs in this region identified in the genetic diagnostic workup of families with SRS or BWS. In addition, these cases will also enlighten the regulatory mechanisms in the two imprinting domains. However, in any case of unclear genotype–phenotype correlation, it should be taken into account that disturbances in other chromosomal regions might be causative for the phenotype.

## Author Contributions

**Anastasia Maria Licata:** writing of the original draft, validation, formal analysis, investigation, visualisation. **Elke Botzenhart** and **Katja Kloth‐Stachnau:** resources. **Thomas Eggermann:** conceptualisation, validation, formal analysis, resources, supervision, project administration, funding acquisition. All authors: Reviewing and editing.

## Funding

This work was supported by the Deutsche Forschungsgemeinschaft (EG115/13).

## Ethics Statement

The study was approved by the ethical committee of the Medical Faculty of the RWTH Aachen (EK303‐18).

## Consent

All patients and their families gave written informed consent.

## Conflicts of Interest

The authors declare no conflicts of interest.

## Supporting information


**Figure S1:** Overview on the literature review process and identified papers. (*These cases include three new CNVs and a grandfather of a recently reported family [11]).

**Table S1:** List of cases and families from the literature and the new cases from this study. Column 1 provides information about the line in Figure 3 in which the cases are included. (dark blue: index patients with duplications, light blue: relatives; dark orange: index patients with deletions, light orange: relatives; dark green: index patients with balanced translocations, light green: relatives).

## Data Availability

The data that support the findings of this study are available on request from the corresponding author. The data are not publicly available due to privacy or ethical restrictions.
