# Supplementary figures and images for: Copy Number Variants in the 11p15.5 Associated Imprinting Disorders: An Attempt to Establish a Genotype–Phenotype Correlation
Source: Clin Genet. 2026 Jan 13;109(6):999–1006. doi: 10.1111/cge.70139 (PMC13167635; doi:10.1111/cge.70139)

## Slide 1
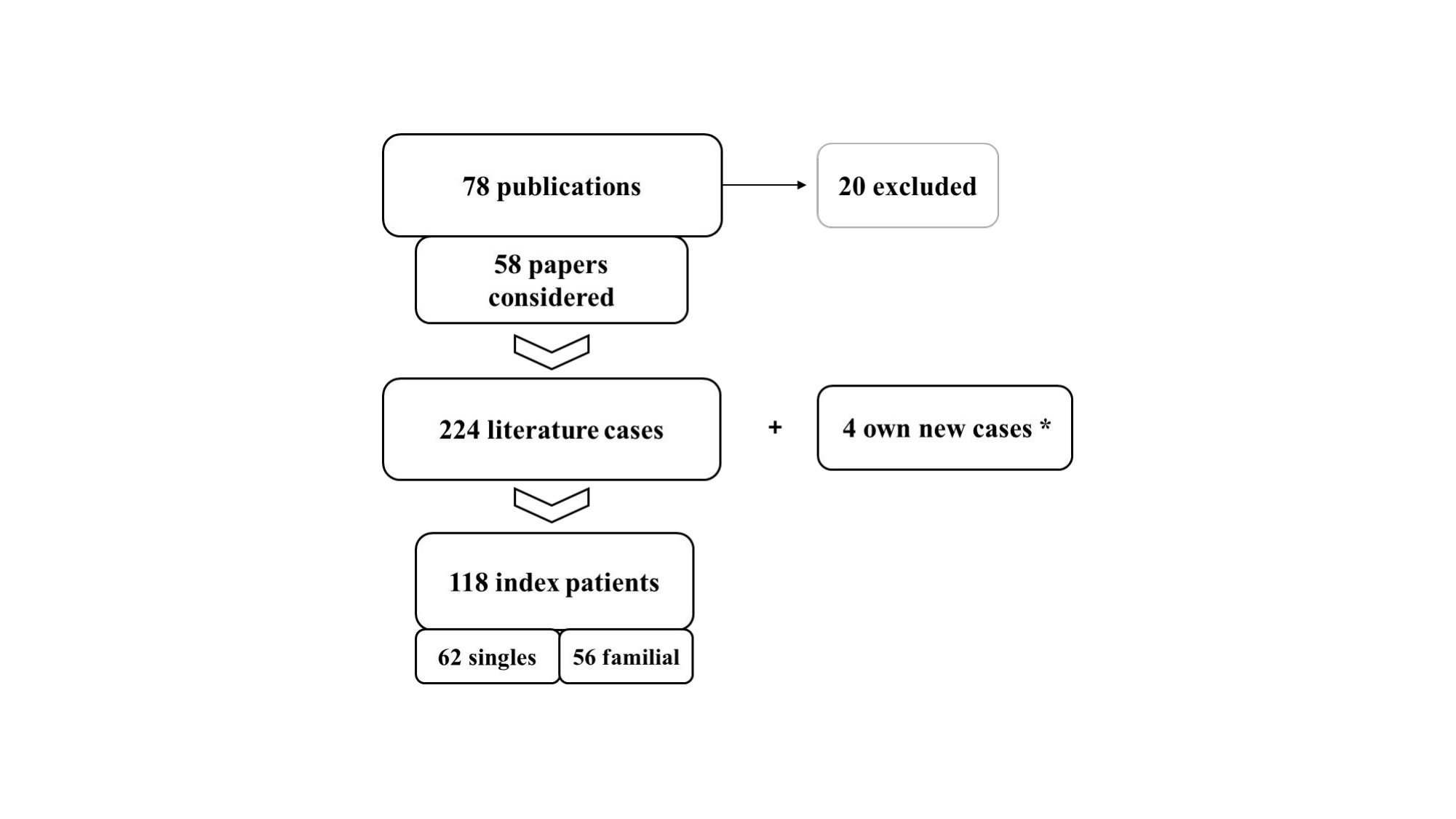

Supplement: Supplementary file 1 — Figure S1: Overview on the literature review process and identified papers. (*These cases include three new CNVs and a grandfather of a recently reported family [11]). [file CGE-109-999-s002.pptx]
